# Supplementary material for: Seminoma and Embryonal Carcinoma Footprints Identified by Analysis of Integrated Genome-Wide Epigenetic and Expression Profiles of Germ Cell Cancer Cell Lines
Source: PLoS One. 2014 Jun 2;9(6):e98330. doi: 10.1371/journal.pone.0098330 (PMC4041891; doi:10.1371/journal.pone.0098330)
Supplement: File S1 — ZIP file containing DMRforPairs output for significant regions. Please start from the html files. (ZIP) [file pone.0098330.s008.zip › figures/6581.pdf]

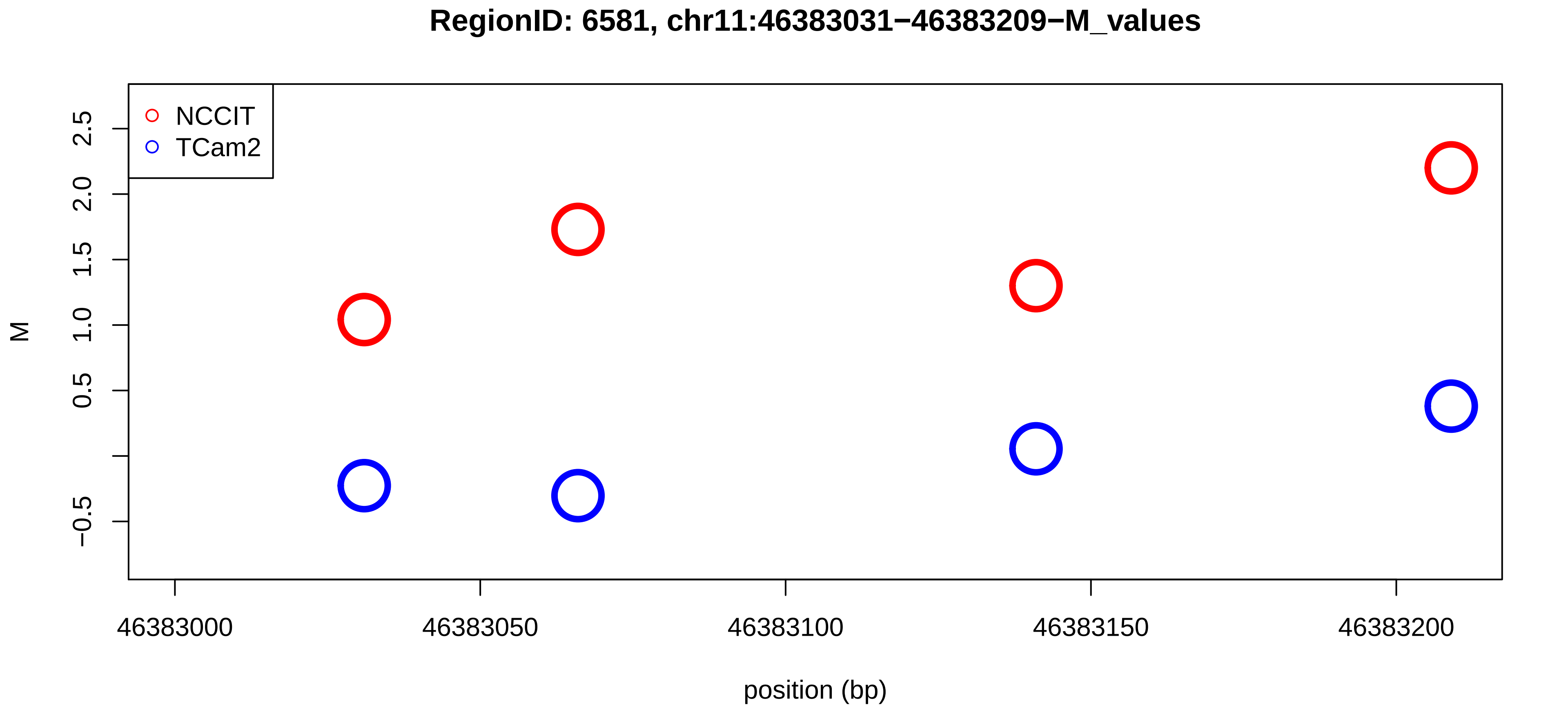
RegionID: 6581, chr11:46383031−46383209−M_values
NCCIT
TCam2
M
2.5
2.0
1.5
1.0
0.5
−0.5
46383000
46383050
46383100
46383150
46383200
position (bp)

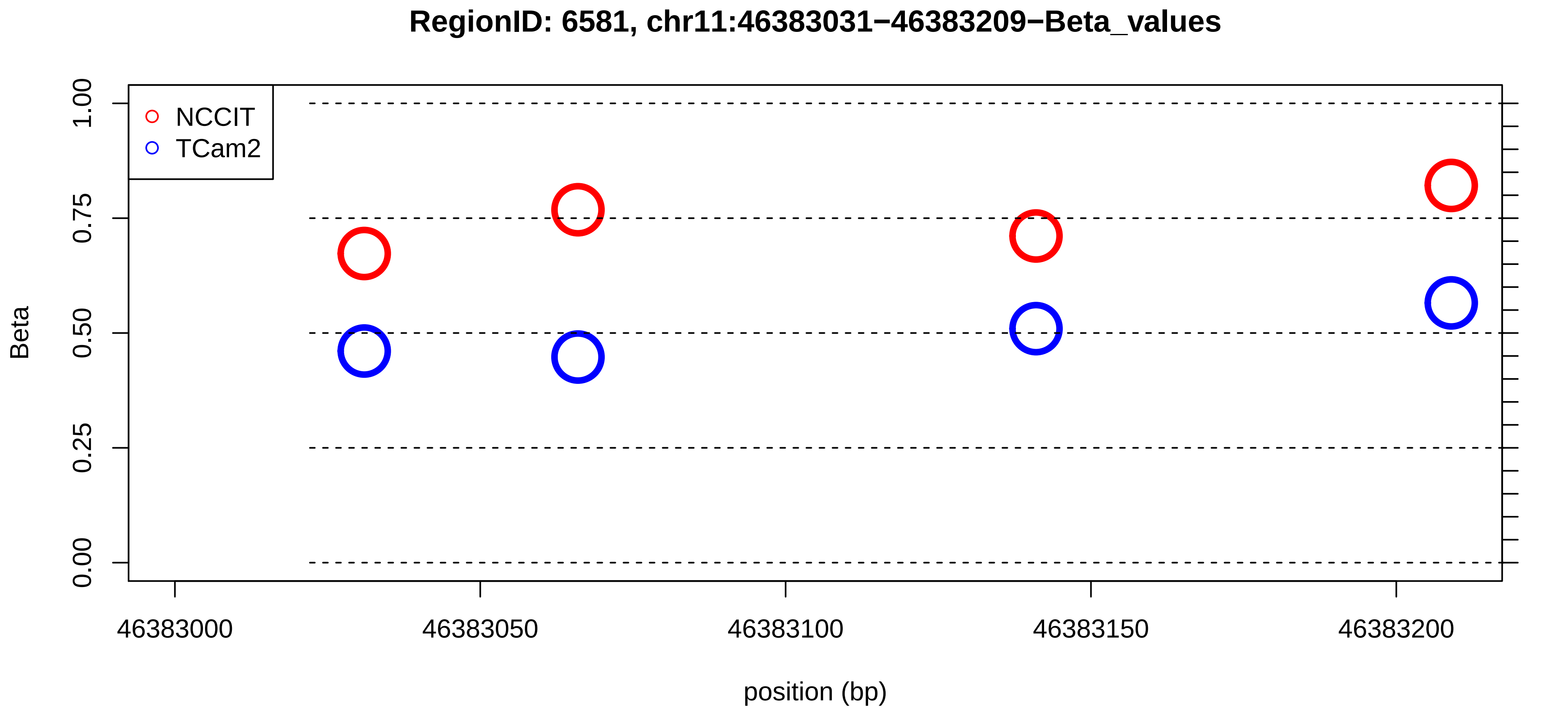
RegionID: 6581, chr11:46383031−46383209−Beta_values
NCCIT
TCam2
Beta
1.00
0.75
0.50
0.25
0.00
46383000
46383050
46383100
46383150
46383200
position (bp)
